# Integrative analyses of potential biomarkers and pathways for non-obstructive azoospermia

**DOI:** 10.3389/fgene.2022.988047

**Published:** 2022-11-24

**Authors:** Yucheng Zhong, Xiaoqing Chen, Jun Zhao, Hao Deng, Xiaohang Li, Zhongju Xie, Bingyu Zhou, Zhuojie Xian, Xiaoqin Li, Guoqun Luo, Huan Li

**Affiliations:** ^1^ Assisted Reproductive Technology Center, Southern Medical University Affiliated Maternal & Child Health Hospital of Foshan, Foshan, China; ^2^ Department of Breast Surgical Oncology, Southern Medical University Affiliated Maternal & Child Health Hospital of Foshan, Foshan, China

**Keywords:** non-obstructive azoospermia, differentially expressed genes, hub genes, bioinformatics, biomarker

## Abstract

**Background:** Non-obstructive azoospermia (NOA) is the most severe form of male infertility. Currently, the molecular mechanisms underlying NOA pathology have not yet been elucidated. Hence, elucidation of the mechanisms of NOA and exploration of potential biomarkers are essential for accurate diagnosis and treatment of this disease. In the present study, we aimed to screen for biomarkers and pathways involved in NOA and reveal their potential molecular mechanisms using integrated bioinformatics.

**Methods:** We downloaded two gene expression datasets from the Gene Expression Omnibus (GEO) database. Differentially expressed genes (DEGs) in NOA and matched the control group tissues were identified using the limma package in R software. Subsequently, Gene ontology (GO), Kyoto Encyclopedia of Genes and Genomes (KEGG), gene set enrichment analysis (GSEA), protein-protein interaction (PPI) network, gene-microRNAs network, and transcription factor (TF)-hub genes regulatory network analyses were performed to identify hub genes and associated pathways. Finally, we conducted immune infiltration analysis using CIBERSORT to evaluate the relationship between the hub genes and the NOA immune infiltration levels.

**Results:** We identified 698 common DEGs, including 87 commonly upregulated and 611 commonly downregulated genes in the two datasets. GO analysis indicated that the most significantly enriched gene was protein polyglycylation, and KEGG pathway analysis revealed that the DEGs were most significantly enriched in taste transduction and pancreatic secretion signaling pathways. GSEA showed that DEGs affected the biological functions of the ribosome, focaladhesion, and protein_expor. We further identified the top 31 hub genes from the PPI network, and friends analysis of hub genes in the PPI network showed that NR4A2 had the highest score. In addition, immune infiltration analysis found that CD8^+^ T cells and plasma cells were significantly correlated with ODF3 expression, whereas naive B cells, plasma cells, monocytes, M2 macrophages, and resting mast cells showed significant variation in the NR4A2 gene expression group, and there were differences in T cell regulatory immune cell infiltration in the FOS gene expression groups.

**Conclusion:** The present study successfully constructed a regulatory network of DEGs between NOA and normal controls and screened three hub genes using integrative bioinformatics analysis. In addition, our results suggest that functional changes in several immune cells in the immune microenvironment may play an important role in spermatogenesis. Our results provide a novel understanding of the molecular mechanisms of NOA and offer potential biomarkers for its diagnosis and treatment.

## 1 Introduction

Over the past few decades, the incidence of infertility has rapidly increased every year. Infertility affects approximately 15% of couples of childbearing age. Azoospermia is the most severe phenotype of male infertility, with approximately 10–15% of infertile men seeking medical attention (Lotti et al., 2014; Tournaye et al., 2017). Some congenital or acquired causes can be found in the vast majority of cases of obstructive azoospermia (OA) (Krausz, 2011; Pan et al., 2018). Non-obstructive azoospermia (NOA), the most severe form of male factor infertility, is characterized by the lack of sperm in the ejaculate and affects about 5–10% of infertile men (Jarow et al., 1989; Lotti et al., 2014; Tournaye et al., 2017). OA is typically characterized by normal spermatogenesis, whereas NOA represents a heterogeneous condition in which spermatogenesis is impaired, including insufficient spermatogenesis and maturation arrest in Sertoli cell-only syndrome (Krausz, 2011; Lotti et al., 2014; Tournaye et al., 2017). Klinefelter syndrome and Y chromosome microdeletions are the most common congenital causes of NOA (Forti et al., 2010; Krausz, 2011; Corona et al., 2017). The etiology of acquired NOA includes torsion, mumps, orchitis, cryptorchidism, and iatrogenic problems (Krausz, 2011; Lotti et al., 2014; Tournaye et al., 2017). For nearly half of the patients with NOA, the etiology remains unknown (Poongothai et al., 2009). Previously, NOA was considered an untreatable condition that required fertilization with donor sperm. With the advent of microdissection testicular sperm extraction (mTESE) and intracytoplasmic sperm injection (ICSI), these techniques have become the first-line treatments for NOA patients (Krausz, 2011; Lotti et al., 2014; Tournaye et al., 2017). Unfortunately, the probability of retrieving sperm is only about 50% in men with NOA owing to partial and heterogeneous preserved focal spermatogenesis (Krausz, 2011; Lotti et al., 2014; Tournaye et al., 2017). Currently, the molecular mechanisms underlying NOA pathology remain unclear. However, this remains an important challenge to solve. Therefore, the identification of genetic abnormalities in patients with NOA is critical.

Several studies have identified potential biomarkers involved in NOA using microarray analysis, weighted gene co-expression network analysis (WGCNA), whole-exome sequencing, and single-cell transcriptome sequencing (scRNA-seq) (Wang et al., 2018; Zheng et al., 2019; Chen et al., 2020; Zhao et al., 2020). However, only a few of these biomarkers are currently used for the diagnosis of NOA. In addition, these studies did not integrate immune infiltration analysis, and the immune system plays an important role in testicular dysfunction and male infertility. Immune infiltration analysis can be used to study the infiltrated immune cells in the testes, to infer and discover the role of immune cells in NOA, and to develop diagnostic and therapeutic targets for the disease. This omission laid the foundation for the present study.

In the present study, we identified differentially expressed genes (DEGs) for NOA by analyzing two mRNA expression profiles downloaded from the Gene Expression Omnibus database (GEO, http://www.ncbi.nlm.nih.gov/geo/). Our results provide a novel understanding of the molecular mechanisms of NOA and offer potential biomarkers for its diagnosis and treatment.

## 2 Materials and methods

### 2.1 Microarray data

**FIGURE 1:**
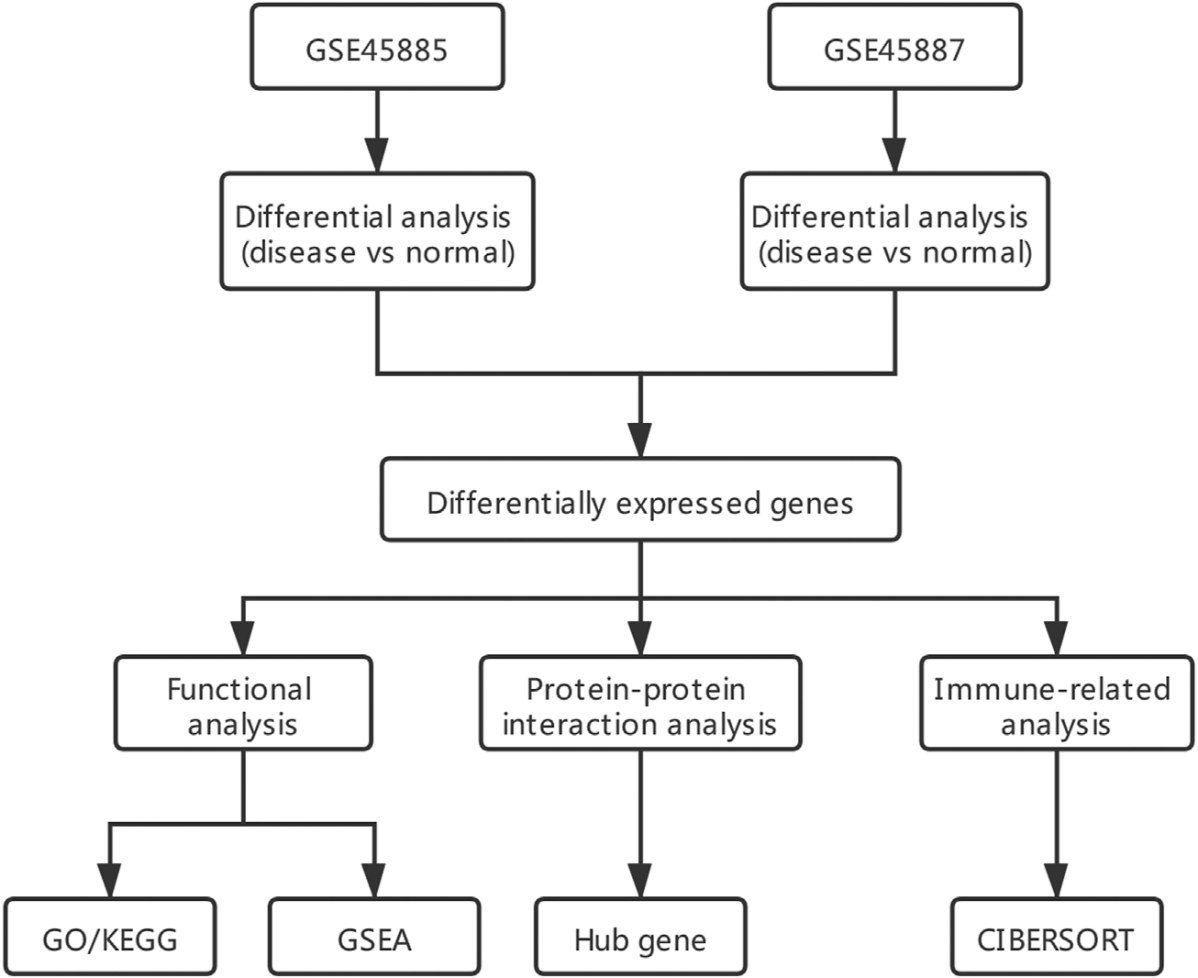
Analysis flow chart. GO/KEGG: Gene ontology (GO) and Kyoto encyclopedia of genes and genomes (KEGG); GSEA: gene set enrichment analysis.

Two gene expression datasets (GSE45885 and GSE45887) (Zhao et al., 2021) related to male NOA were derived from GEO (GPL6244 [HuGene-1_0-st] Affymetrix Human Gene 1.0 ST Array [transcript (gene) version]) (Chicco, 2022) using the R package GEOquery (Hunt et al., 2022) of R software program. The data type was expression profiling by array, and the species was *Homo sapiens*. Twenty-seven NOA and four normal control samples were obtained from GSE45885, and GSE45887 contained 16 NOA and four normal control samples. The characteristics of GSE45885 and GSE45887 patients can be seen in Supplementary Tables S1, S2. The raw data from the GSE45885 and GSE45887 datasets were normalized using the limma package (Liu et al., 2021). The data analysis process can be seen in Figure 1.

### 2.2 Identification of differentially expressed genes (DEGs)

DEGs with the threshold criterion of |log2FC| >1 and *p* < 0.05 from NOA and normal control samples in GSE45885 and GSE45887 datasets were screened with the limma package (Walker, 2009). Subsequently, heatmaps and volcano plots of DEGs from each dataset were plotted using the pheatmap package (Ning et al., 2022) and ggplot2 package (Gustavsson et al., 2022) in the R analysis platform.

### 2.3 Gene ontology (GO) and kyoto encyclopedia of genes and genomes (KEGG) enrichment analyses of DEGs

GO analysis is a common method for annotating and analyzing the biological processes of genes, including biological processes (BPs), molecular functions (MFs), and cellular components (CCs) (Ge et al., 2020). KEGG is a widely used database that stores information on genomes, biological pathways, diseases, and drugs (Kanehisa et al., 2021). We performed biological analyses using clusterProiler (Wu et al., 2021) in the R software statistical analysis platform (significant at *p* < 0.05) to visualize the GO terms and KEGG pathway enrichment analysis of DEGs from NOA and normal control samples.

### 2.4 Gene set enrichment analysis (GSEA)

GSEA determines whether a set of predefined genes shows statistically significant, concordant differences between related phenotypes to screen for significant differential biological functions (Nguyen et al., 2021). We obtained the “C2. kegg.v7.4. symbols” gene set from MSigDB (Liberzon et al., 2015) for GSEA of the two datasets. Furthermore, GSEA was automatically completed and visualized using clusterProiler (Wu et al., 2021) in R software, and statistical significance was set at *p* < 0.05.

### 2.5 Construction of protein-protein interaction (PPI) network

PPI (Das and Mitra, 2021) networks are composed of individual proteins that interact with each other, and are involved in various aspects of biological signal transmission, gene expression regulation, energy and substance metabolism, and cell cycle regulation. Systematic analysis of functional interactions between proteins is of great significance for comprehending the principle of proteins in biological systems, the reaction mechanism of biological signals, and the mechanisms of generation or development of diseases under special physiological states, as well as the functional relationships between proteins.

The STRING (Szklarczyk et al., 2021) database currently covers 9.6 million proteins and 13.8 million protein-protein interactions from 5,090 organisms and contains known and predicted protein-protein interactions. The results of the STRING database were derived from experimental data, PubMed abstract texts, other database data, and bioinformatics methods. In this study, a PPI network of DEGs of NOA was constructed using the STRING database and visualized using the Cytoscape (Puig et al., 2020) software.

### 2.6 Construction of miRNA-hub gene and transcription factor-hub gene regulatory networks

Hub genes were identified using GOSemSim (Kamran and Naveed, 2022) in R software. In addition, the functional similarity between proteins was evaluated using the geometric mean of semantic acquaintance in CCs and MF using GOSemSim in R software. We also analyzed hub genes using the NetworkAnalyst database (https://www.networkanalyst.ca/NetworkAnalyst/home.xhtml) and constructed miRNA-hub gene interaction and transcription factor-hub gene networks.

### 2.7 Immune infiltration analysis by CIBERSORT

CIBERSORT (Le et al., 2021) is a deconvolution algorithm for the expression matrix of immune cell subtypes based on linear support vector regression to estimate the abundance of immune cell infiltration in a mixed-cell population using RNA-seq data. We analyzed immune cell infiltration and the proportion of NOA tissue using CIBERSORT and conducted a correlation analysis of related immune cells by obtaining the hub gene to evaluate the relationship between the hub gene and NOA immune infiltration levels.

### 2.8 Statistical analysis

R version 4.0.2 software (https://cran.r-project.org/) was used to conduct the statistical analyses. For the comparison of the two groups of continuous variables, the statistical significance of the normally distributed variables was estimated using the independent Student’s t-test, and the differences between the non-normally distributed variables were analyzed using the Mann-Whitney *U* test. All statistical *p*-values were bilateral, and a *p*-value of <0.05 was considered statistically significant.

## 3 Results

### 3.1 Identification of DEGs in NOA

**FIGURE 2:**
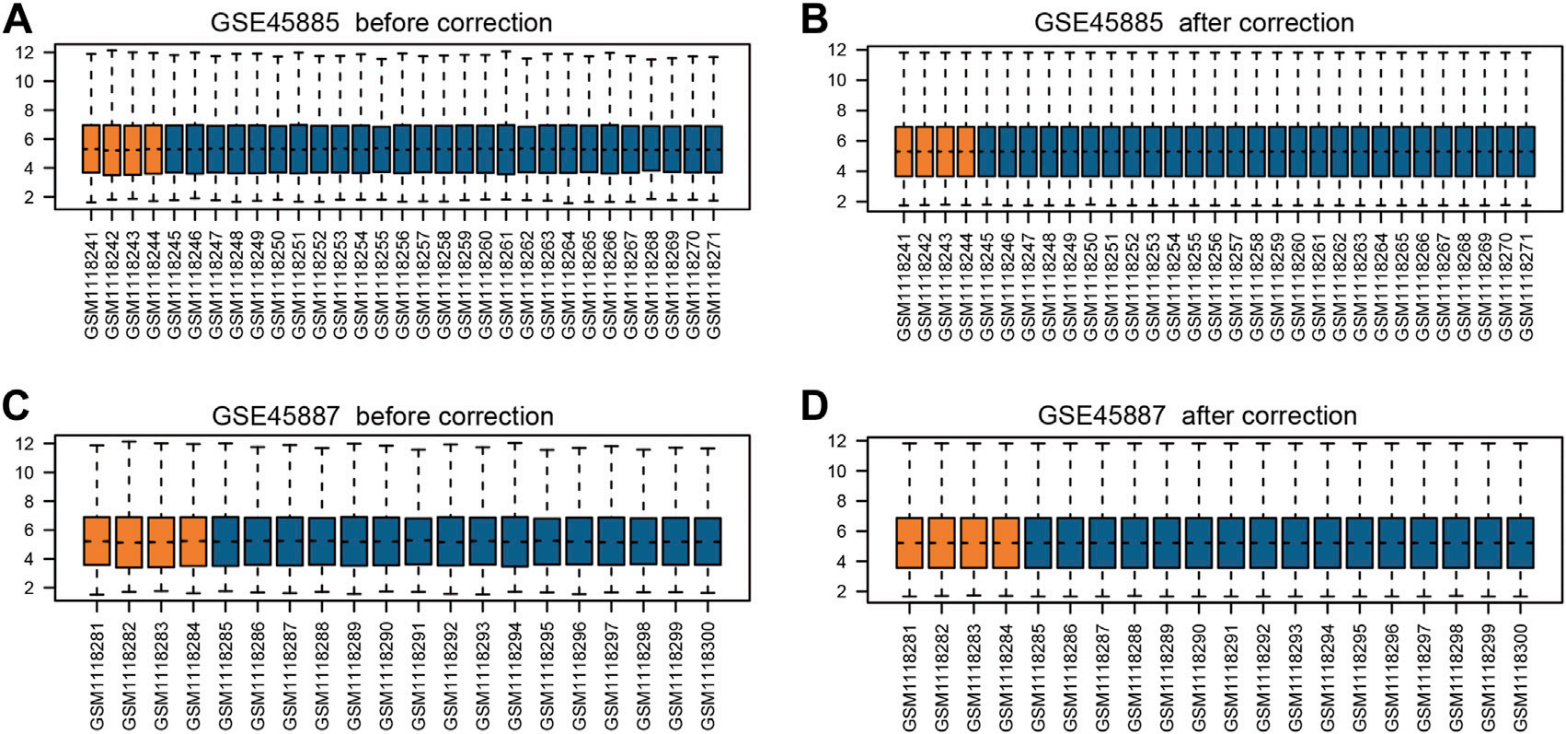
Box plots of gene expression data before and after normalization. **(A)** GSE45885 before correction, **(B)** GSE45885 after correction, **(C)** GSE45887 before correction, **(D)** GSE45887 after correction. The x-axis label represents the sample symbol and the y-axis label represents the gene expression values. The orange bar represents the data of normal control samples and the blue bar represents the data of NOA samples.

**FIGURE 3:**
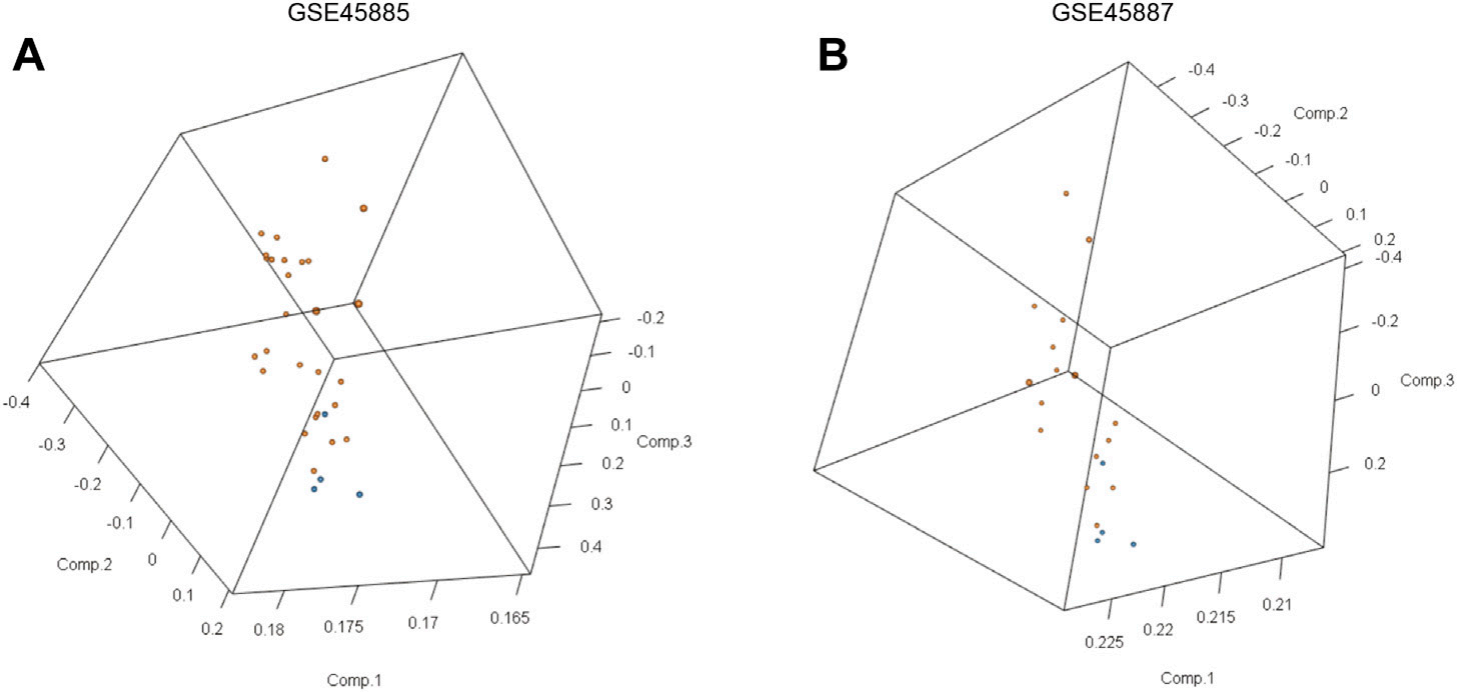
Principal component analyses (PCA) of gene expression between the NOA group and control group in GSE45885 and GSE45887. **(A)** 3D-PCA data distribution of GSE45885, **(B)** 3D-PCA data distribution of GSE45887. The orange point represents the data of normal control samples and the blue point represents the data of NOA samples.

First, we obtained the corresponding data using the R package GEOquery package and standardized the original datasets using the limma package (Figures 2A–D). In order to further judge whether the data set samples have obvious overall differences in expression profiles, we performed PCA analysis and found that the disease group and control group samples had obvious overall differences (Figures 3A,B).

**FIGURE 4:**
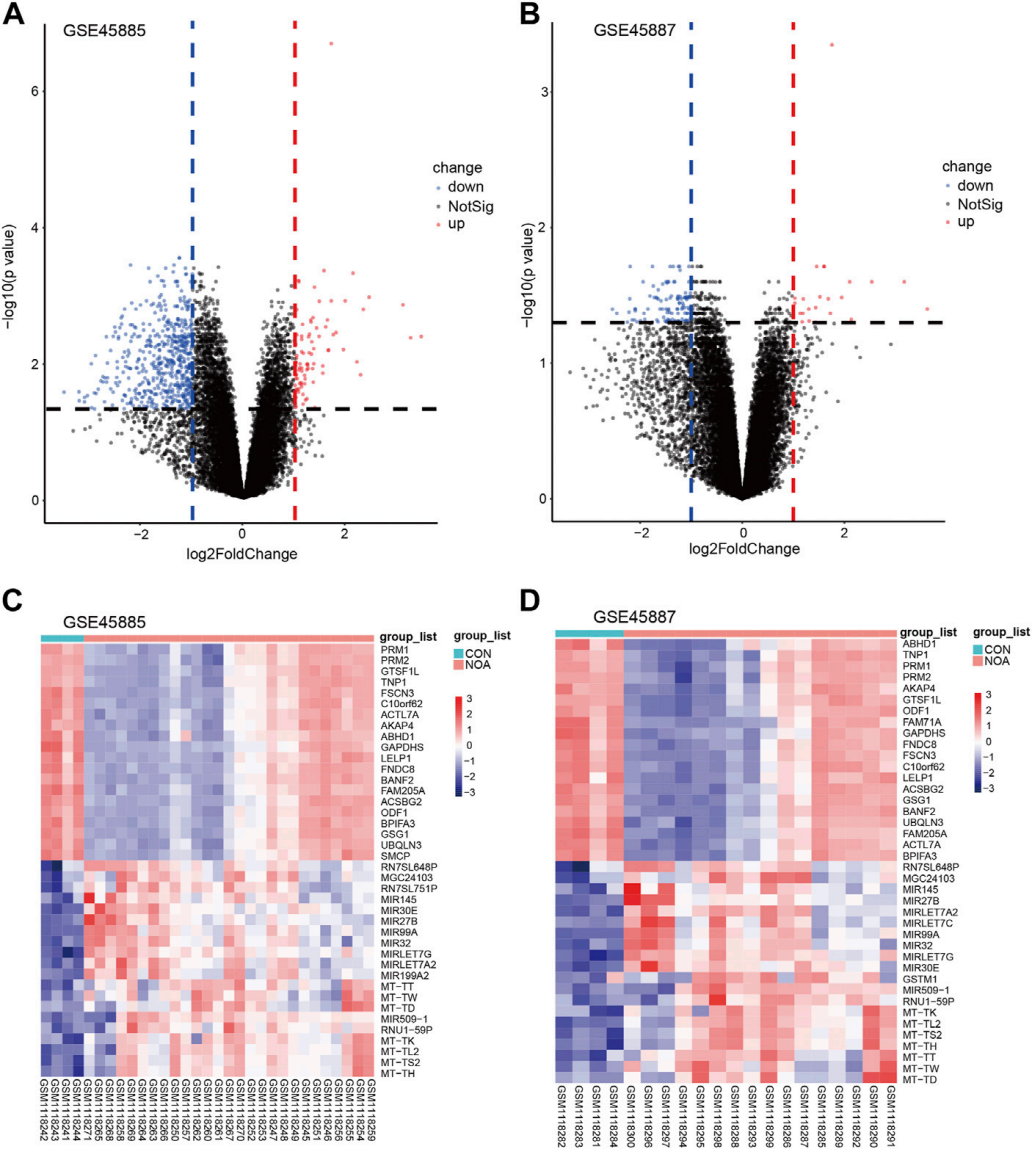
Volcano plots and heatmap of DEGs. **(A)** Volcano plots of GSE45885, **(B)** volcano plots of GSE45887. The x-axis label represents log2FoldChange and the y-axis label represents–log10 (adjusted *p*-value). Data points in red represent upregulated, and green represent downregulated genes. No significantly changed genes are marked as gray points. Heatmap of the top 40 DEGs screened by limma package in R software. **(C)** Heatmap of GSE45885, **(D)** heatmap of GSE45887. Red areas represent highly expressed genes and blue areas represent lowly expressed genes in NOA. Darker color indicates the higher multiple of DEGs. DEGs: differentially expressed genes; NOA: non-obstructive azoospermia.

Next, we used the limma package to perform differential analysis, and the results are as follows: the combined GEO dataset (Combined Datasets) has a total of 698 differentially expressed genes (DEGs) that satisfy the threshold of |logFC| > 0 and adj.*p* < 0.05, under this threshold, 70 up-regulated genes (logFC >0 and adj. *p* < 0.05) and 507 down-regulated genes (logFC <0 and adj. *p* < 0.05) in the GSE45885 dataset (Figure 4A), and utilized the top 40 differentially expressed genes A classification heat map was drawn (Figure 4B); there were 17 up-regulated genes (logFC >0 and adj. *p* < 0.05) and 104 down-regulated genes (logFC <0 and adj. *p* < 0.05) in the GSE45887 dataset. The 40 differentially expressed genes were classified as heatmaps (Figure 4D). The top 40 differential genes obtained are: PRM1, PRM2, GTSF1L, TNP1, FSCN3, C19orf62, ACTL7A, AKAP4, ABHD1, GAPDHS, LELP1, FNDC8, BANF2, FAM205A, ACSBG2, ODF1, BPIFA3, GSG1, UBQLN3, SMCP, RN7SL648P, MGC24103, RN7SL751P, MIR145, MIR30E, MIR27B, MIR99A, MIR32, MIRLET7G, MIRLET7A2, MIR199A2, MT-TT, MT-TW, MT-TD, MIR509-1, RNU1-59P, MT-TK, MT-TL2, MT -TS2, MT-TH (Figures 4C,D).

### 3.2 PPI network construction and hub gene identification

**FIGURE 5:**
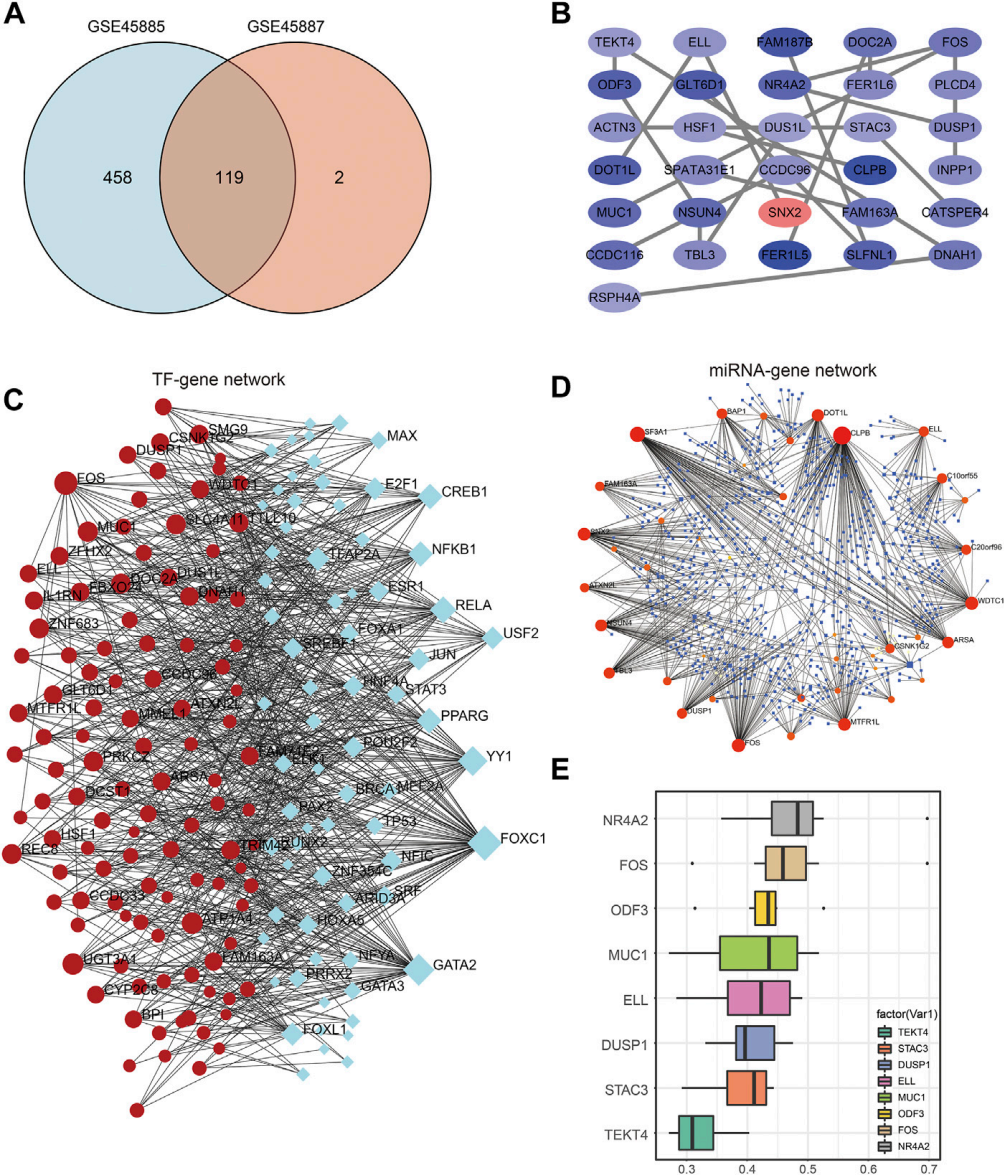
PPI network construction and hub gene regulatory network. **(A)** Venn diagram of co-expressed DEGs from GSE45885and GSE45887. **(B)** DEG-related PPI networks of NOA. Red nodes represent highly expressed genes and blue nodes represent lowly expressed genes in NOA. **(C)** DEG-related TF-mRNA networks of NOA. **(D)** DEG-related miRNA-mRNA networks of NOA. **(E)** Friend analysis of hub gene in PPI network. DEGs: differentially expressed genes; NOA: non-obstructive azoospermia; PPI: protein-protein interaction.

We intersected the differentially expressed genes from the analysis of the two datasets and visualized them using a Venn diagram, resulting in 119 differentially expressed genes (Figure 5A). Then, we conducted PPI (Protein-Protein Interaction Networks, PPI) analysis on 119 differential genes through the STRING database, and imported the protein interaction results into Cytoscape for visualization and drawing, and found that 31 genes have strong similarities of biological functions (Figure 5B), are: TEKT4, ODF3, ACTN3, DOT1L, MUC1, CCDC116, RSPH4A, ELL, GLT6D1, HSF1, SPATA31E1, NSUN4, TBL3, FAM187B, NR4A2, DUS1L, CCDC96, SNX2, FER1L5, DOC2A, FER1L6, STAC3, CLPB, FAM163A, SLFNL1, FOS, PLCD4, DUSP1, INPP1, CATSPER4, DNAH1.

Then, the networkAnalyst database was used to analyze these 119 genes, and jointly used the JASPAR database (Zheng et al., 2019) and miRTarBase v8.0 (Chen et al., 2020) to analyze the transcription factors of differential genes and their possible binding miRNAs. The results of the analysis were then imported into Cytoscape for visualization, displaying the transcription factor-differential gene network (Figure 5C) and the differential gene-miRNA network (Figure 5D). Figure 5E shows the friends analysis results of the hub gene. The top 8 genes with scores are NR4A2, FOS, COF3, MUC1, ELL, DUSP1, STAC3, TEKT4, among which NR4A2 has the highest score, which may play an important role in the occurrence of NOA effect.

### 3.3 KEGG and GO enrichment analyses

**FIGURE 6:**
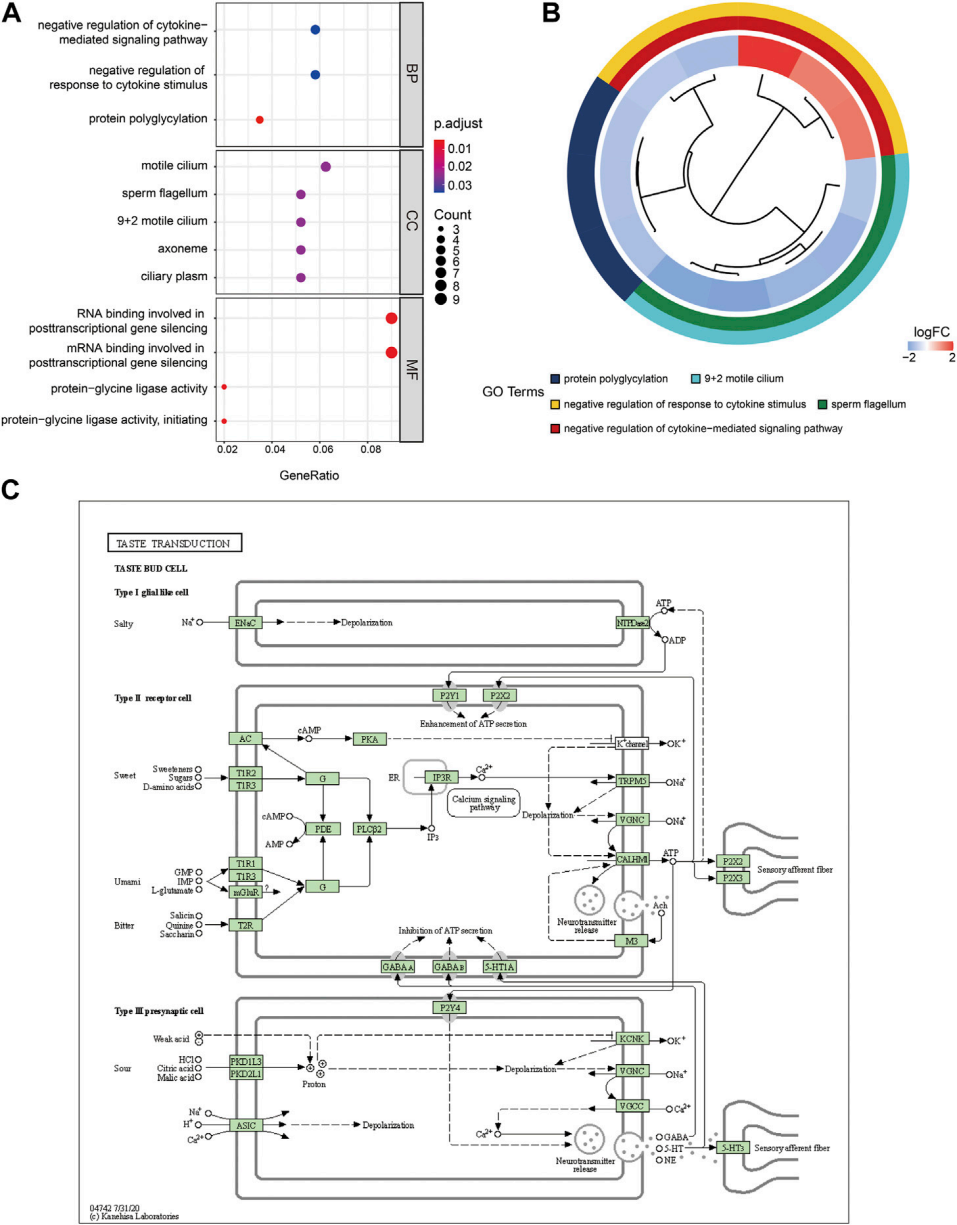
GO enrichment and KEGG pathway analysis of DEGs. **(A)** GO enrichment result of DEGs. The x-axis label represents gene ratio and y-axis label represents GO terms. The color indicates GO terms, red indicates activated and blue indicates inhibited. The size of circle represents gene count. Different colors of circles represent different adjusted *p* values. **(B)** GOplot results combined with gene expression logFC. **(C)** The most significant enrichment signaling pathway was hSA04742: taste transduction. GO: Gene ontology; KEGG: Kyoto Encyclopedia of Genes and Genomes; MF: molecular function; BP: biological processes; CC: cell composition.

To investigate the relationship between the DEGs of NOA and BCs, MFs, CCs, biological pathways, and diseases, functional and pathway enrichment analyses were performed. Regarding BPs of GO analysis, DEGs of NOA were significantly enriched in protein polyglycylation, negative regulation of cytokine-mediated signaling pathways, and negative regulation of response to cytokine stimulus. For CCs, DEGs of NOA were significantly enriched in sperm flagellum, 9 + 2 motile cilium, axoneme, ciliary plasm, and motile cilium. Changes in MFs of hub genes were mainly enriched in RNA binding involved in posttranscriptional gene silencing, mRNA binding involved in posttranscriptional gene silencing, protein-glycine ligase activity, and protein-glycine ligase activity. Additionally, using the logFC value of genes, we visualized the changes in gene expression and the relationship between functions using GOplot software (Figure 6B). KEGG pathway enrichment demonstrates that the DEGs of NOA were primarily enriched in the taste transduction and pancreatic secretion signaling pathway Supplementary Table S3, and the most significant enrichment signaling pathway, hSA04742: Taste transduction, is shown in Figure 6C.

### 3.4 GSEA and gene set variation analysis (GSVA)

**FIGURE 7:**
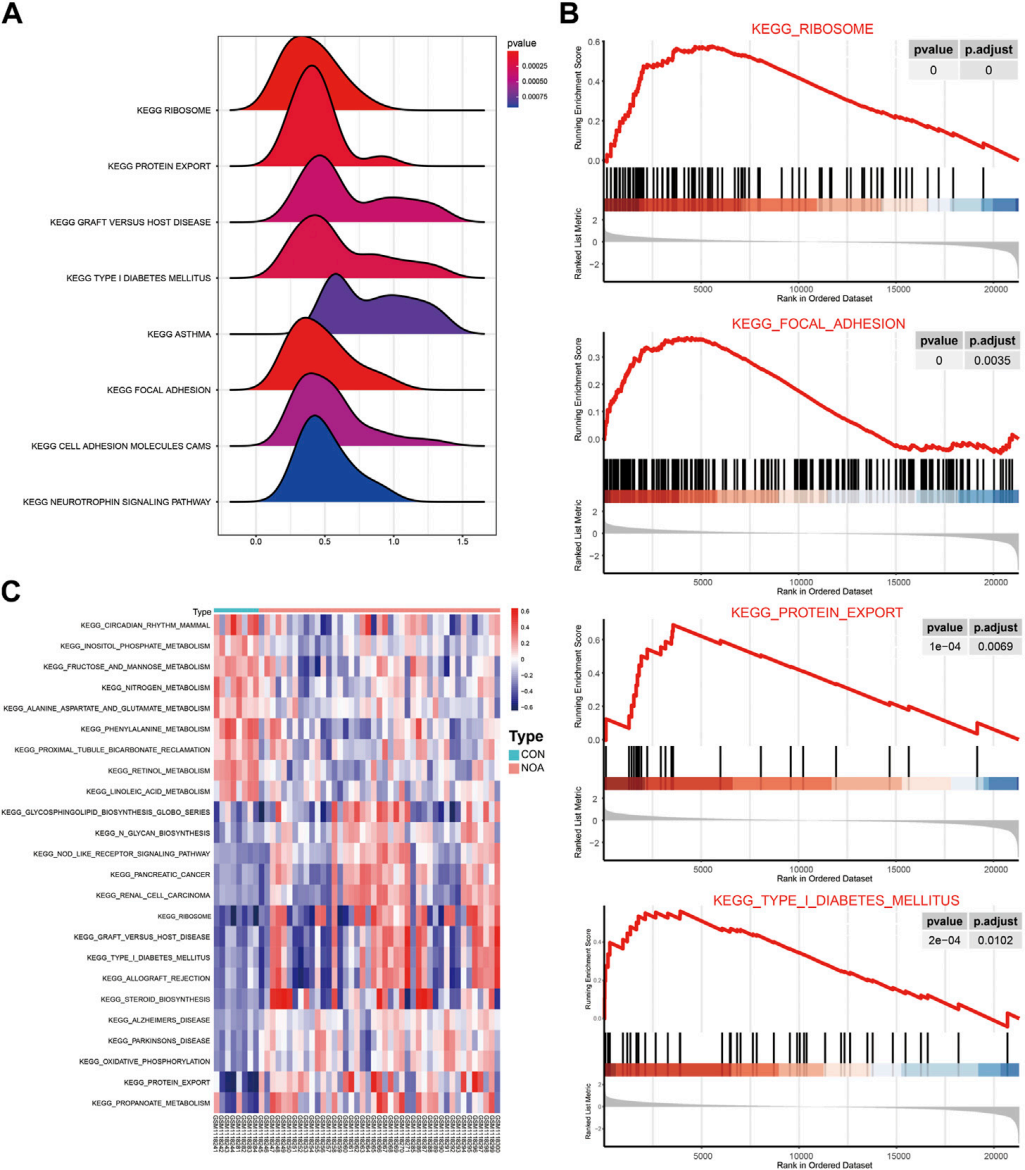
GSEA and GSVA. **(A)** Results of GSEA are presented by ridge maps. The x-axis label represents gene ratio and y-axis label represents KEGG pathway. **(B)** Top four most significant enriched gene sets in NOA: ribosome signaling pathway; focal adhesion signaling pathway; protein expor signaling pathway; type I diabetes mellitus signaling pathway. **(C)** Results of GSVA were visualized with heatmaps. Red indicates activated and blue indicates inhibited. GSEA: Gene Set Enrichment Analysis; GSVA: Gene Set Variation Analysis.

GSEA was performed to determine the effect of gene expression on NOA, and the results show that the DEGs affected the biological functions of the ribosome, focaladhesion, and protein_expor (Figures 7A,B). To evaluate the enrichment of different metabolic pathways in different samples, we analyzed the expression levels of genes in different samples that converted them into gene sets in different samples using GSVA, and the enrichment was visualized using the pheatmap package (Figure 7C). We found that sample grouping could distinguish the results of the GSEA.

### 3.5 Immune infiltration analysis

To assess the level of immune infiltration in male non-obstructive azoospermia tissue, we used the CIBERSORT algorithm to calculate the degree of infiltration of 22 types of immune cells in the tissue.

**FIGURE 8:**
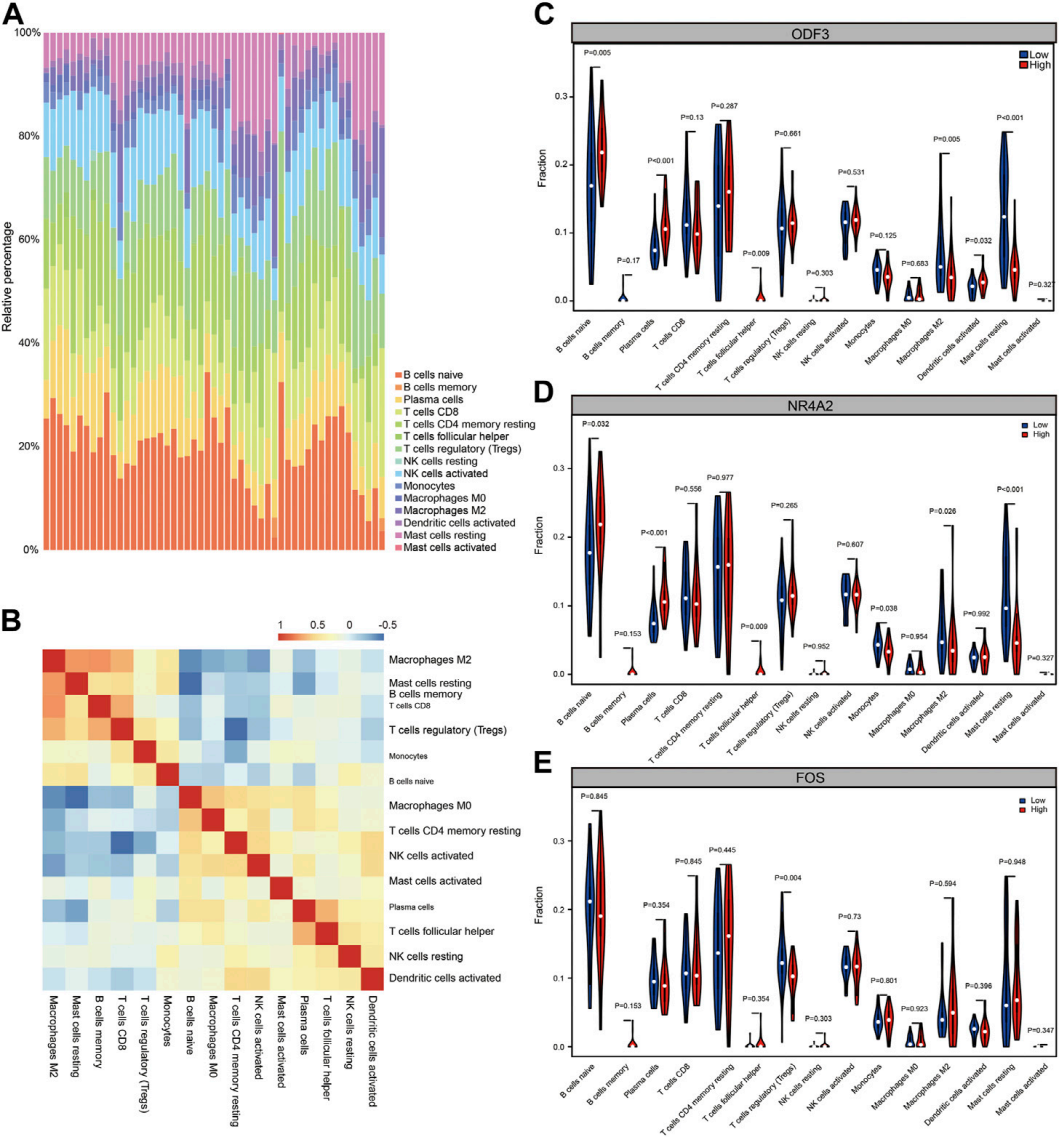
Immune infiltration analysis. **(A)** CIBERSORT algorithm analysis of immune cell infiltration panorama. Different colors represent different immune cell subsets. **(B)** CIBERSORT algorithm analysis of immune cell infiltration panorama correlation heat map. Red represents positive correlation and blue represents negative correlation. **(C)** Functional correlation between ODF3 expression in NOA and immune cells. **(D)** Functional correlation between NR4A2 expression in NOA and immune cells. **(E)** Functional correlation between FOS expression in NOA and immune cells.

Using the wilcox.test algorithm to analyze and filter out immune cells with low expression abundance, a total of 15 immune cells were included, including B cells naive, B cells memory, Plasma cells, T cells CD8, T cells CD4 memory resting, T cells follicular helper, T cells regulatory (Tregs), NK cells resting, NK cells activated, Monocytes, Macrophages M0, Macrophages M2, Dendritic cells activated, Mast cells resting, Mast cells activated, and draw a panorama of immune cell entry in NOA (Figure 8A). Next, the correlation between individual immune cells in both datasets was assessed (Figure 8B). To assess the functional correlation between key genes and immune cells in male non-obstructive azoospermia, we selected the top three hub genes for analysis.

According to the expression value of the hub gene, the samples were divided into high expression group and low expression group, and calculated the difference of immune cell infiltration levels between the high and low expression groups. As shown in Figures 8C–E, in the disease group and the ODF3 high and low expression group, there were differences in the immune cell infiltration of B cells naïve, plasma cells, Macrophages M2, Dendritic cells activated, and Mast cells resting, among which Macrophages M2 and Mast cells resting were low. Expression genes, plasma cells, B cells naïve, and Dendritic cells activated are highly expressed genes; however, in the high and low expression groups of NR4A2 gene, there are differences in the immune cell infiltration of B cells naive, Plasma cells, Monocytes, Macrophages M2, and Mast cells resting. The expressed genes are B cells naïve, Plasma cells, and the low-expressed genes are Monocytes, Macrophages M2, Mast cells resting, and there are differences in the infiltration of T cells regulatory (Tregs) immune cells in the high and low expression groups of FOS genes, which are low-expressed genes. It was statistically significant (*p* < 0.05, statistically significant; *p* < 0.01, highly statistically significant; *p* < 0.001, extremely statistically significant). The above results indicate that the functional changes of several immune cells may play an important role in the immune microenvironment of NOA patients.

## 4 Discussion

Spermatogenesis is a complex and subtle process characterized by three specific functional phases: mitotic proliferation and expansion, meiosis, and spermiogenesis (Neto et al., 2016). Genetic mutations play an important role in NOA (Peña et al., 2020). Compared to that of OA, the pathogenesis of NOA is more complex and difficult to understand. However, there are currently no specific therapeutic targets for NOA. Thus, it is of great significance to construct a molecular regulatory network of NOA to search for therapeutic targets. In this study, we integrated the GSE45885 and GSE45887 datasets and identified 527 and 121 common DEGs, respectively, using bioinformatics methods, among which 119 DEGs were at the intersection of the two datasets and 31 genes had similar biological functions. To further investigate the molecular mechanism of the pathogenesis of NOA, we analyzed the transcription factors of 119 DEGs and their possible binding miRNAs and successfully identified three hub genes (NR4A2, FOS, and ODF3). KEGG and GO enrichment analyses of DEGs showed that the DEGs of NOA were mainly enriched in taste transduction and pancreatic secretion signaling pathways. Moreover, the three most important hub genes (NR4A2, FOS, and ODF3) were analyzed for immune cell infiltration in NOA testicular tissue, suggesting that changes in the function of several immune cells in the immune microenvironment may play an important role in spermatogenesis. These results suggest that NR4A2, FOS, and ODF3 are potential biomarkers for the diagnosis and treatment of NOA.

NR4A2 belongs to the nuclear receptor superfamily and is involved in multiple BPs including proliferation, metabolism, immunity, cellular stress, apoptosis, and DNA repair (Safe et al., 2016). In addition to Nr4a proteins, which are known transcription factors, they are also important receptors of hormones (Ke et al., 2004). Testosterone and dihydrotestosterone secreted from Leydig cells are crucial for the initiation and maintenance of spermatogenesis (Walker, 2009). Dysfunction of Leydig cells contributes to testicular spermatogenesis disorders in OA patients (Walker, 2009). A previous study reported that NR4A2 plays an essential role in Leydig cells, and can regulate the transcription of steroidogenic acute regulatory protein (StAR) or 3β-hydroxysteroid dehydrogenase (3β-HSD) in Leydig cells (Martin and Tremblay, 2010; Hu et al., 2018). Dai et al. (2021), (Martin et al., 2008) investigated the expression patterns, distribution, and functions of NR4A2 in male Tianzhu white yaks and found that NR4A2 is involved in the regulation of male yak reproduction, especially steroid hormones and androgen metabolism. Our study further indicates that NR4A2 might play an essential role in spermatogenesis, and the detailed regulatory mechanism of NR4A2 in the spermatogenesis of patients with NOA needs to be further studied. FOS, also known as the c-fos gene, is a transcription factor. One study showed that the expression of c-fos mRNA is appreciably lower and ERβ mRNA is higher in the testes of men with NOA than in those with OA, which suggests that c-fos transcriptional activity is associated with spermatogenesis. However, few studies have focused on c-Fos transcriptional activity during spermatogenesis, and the detailed mechanisms are unclear. The outer dense fiber (ODF) protein is a keratin that includes ODF1, ODF2, and ODF3 (Araújo et al., 2009). The tail is an important structure for proper sperm function. Studies have demonstrated that ODF proteins are the main cytoskeletal structures of the sperm tail and are preferentially expressed during mammalian spermiogenesis (Kierszenbaum, 2002; Petersen et al., 2002). Among them, ODF3 is transcribed in the testes, more specifically in spermatids, and is involved in sperm tail formation (Petersen et al., 2002; Sarkar et al., 2022). Unfortunately, there is little evidence that ODF3 is an important trigger for the pathophysiological process of NOA.

We conducted KEGG and GO enrichment analyses of DEGs and found that the DEGs of NOA were mainly enriched in taste transduction and pancreatic secretion signaling pathways. A recent study showed that olfactory receptor 2 is present in vascular macrophages (Orecchioni et al., 2022). However, no studies have reported the involvement of these pathways in spermatogenesis. Whether these pathways are involved in spermatogenesis needs to be verified using larger sample sequencing and experiments.

A previous study has confirmed that inflammation in the reproductive tract contributes to testicular dysfunction and male infertility (Hedger, 2011). Therefore, we performed immune infiltration analysis and found that immune cells are involved in the pathophysiological process of NOA. One study reported that T cell subsets are essential for intact spermatogenesis and may be targets for the treatment of chronic orchitis and immune infertility (Gong et al., 2020). Suppressor T cells predominate in patients with obstructive azoospermia, whereas T cells of the helper phenotype predominate in patients with unilateral testicular obstruction (el-Demiry et al., 1987), but T cells and their subset distribution in the testis of NOA are unknown. In addition, several studies have suggested that B cells and macrophages contribute to spermatogenesis (Cabas et al., 2011; de Oliveira et al., 2021), similar to our results. Dong et al. (2021) also found that macrophages are the most important immune cells in NOA by immune infiltration analysis. All these studies showed that immune cells play an important role in NOA, and the mechanism by which immune cells impact spermatogenesis should be further studied.

Although we obtained several key findings in the present study, there are still some limitations. First, although we analyzed two datasets with 51 patients, the effects of race and region on the findings were not observed. We look forward to verifying our results using a larger, real-world sample size. Second, our analysis was based on data from a public database and we did not conduct experiments to verify our results. Third, the hub genes, pathway enrichment, and results of immune infiltration analysis were not validated by external datasets or clinical samples. Fourth, we must admit that WGCNA is a better analytical method, while DEGs is the more common method to screen for differential genes. We believe that the analysis method combining WGCNA and DEGs will make the results more rigorous and credible, which is what we need to work on in the future. Finally, owing to the lack of patient prognosis information in the dataset, genes related to patient prognosis were not screened out.

In conclusion, the present study successfully constructed a regulatory network of DEGs between NOA and normal controls and screened three hub genes using integrative bioinformatics analysis. In addition, our results suggest that functional changes in several immune cells in the immune microenvironment may play an important role in spermatogenesis. Our results provide a novel understanding of the molecular mechanisms of NOA and offer potential biomarkers for its diagnosis and treatment.

## Data Availability

Publicly available datasets were analyzed in this study. The names of the repository/repositories and accession number(s) can be found in the article/Supplementary Material.
