# Supplementary material for: Integrative analyses of potential biomarkers and pathways for non-obstructive azoospermia
Source: Front Genet. 2022 Nov 24;13:988047. doi: 10.3389/fgene.2022.988047 (PMC9730279; doi:10.3389/fgene.2022.988047)
Supplement: Supplementary file 2 [file Table1.DOCX]

**Table S1 Clinical and demographic characteristics of the patients from GSE45885**

|  | All subjects | NOA | Controls |
| --- | --- | --- | --- |
| Patients | 31 | 27 | 4 |
| Testis region |  |  |  |
| Right | 15 | 15 | 0 |
| Left | 11 | 11 | 0 |
| N/A | 4 | 0 | 4 |
| Pull-right and left | 1 | 1 | 1 |
| Histopatological description |  |  |  |
| Full spermatogenesis | 4 | 0 | 4 |
| Meiotic arrest  Postmeiotic arrest  Sertoil Cell Only-Syndrome | 7  13  7 | 7  13  7 | 0  0  0 |
| Age(years) |  | 41±13 | 39±25 |

“Non-obstructive azoospermia=NOA”
